# Supplementary material for: High levels of TFAM repress mammalian mitochondrial DNA transcription in vivo
Source: Life Sci Alliance. 2021 Aug 30;4(11):e202101034. doi: 10.26508/lsa.202101034 (PMC8408345; doi:10.26508/lsa.202101034)

Figure 1B

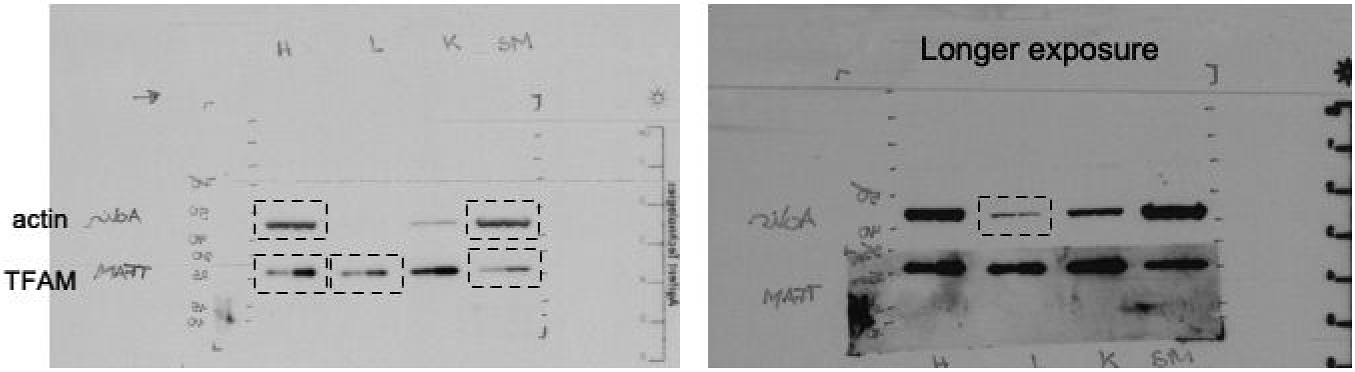

Figure 1D

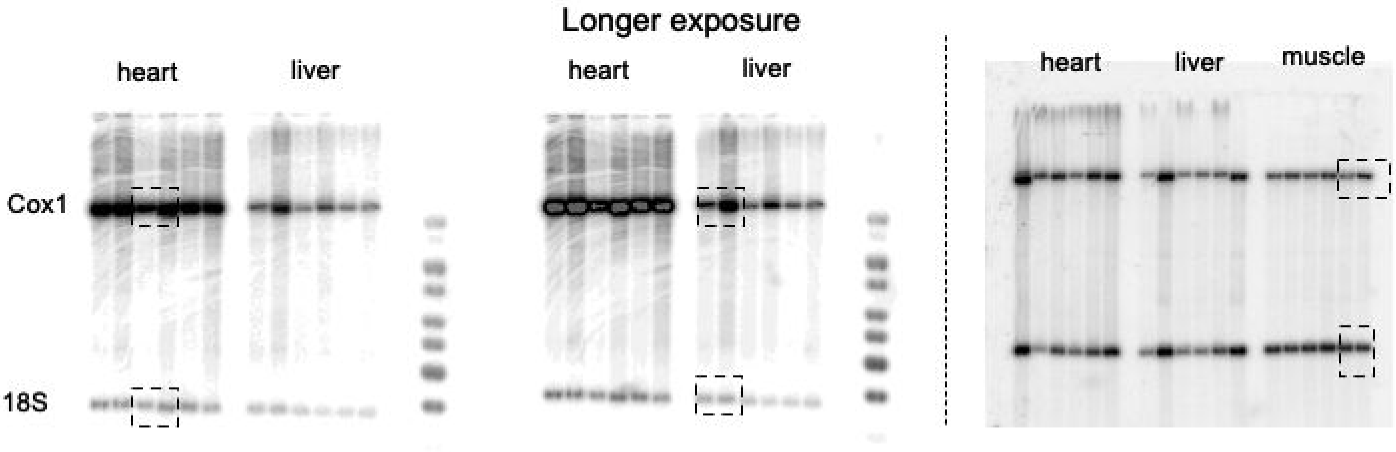

Figure 1F

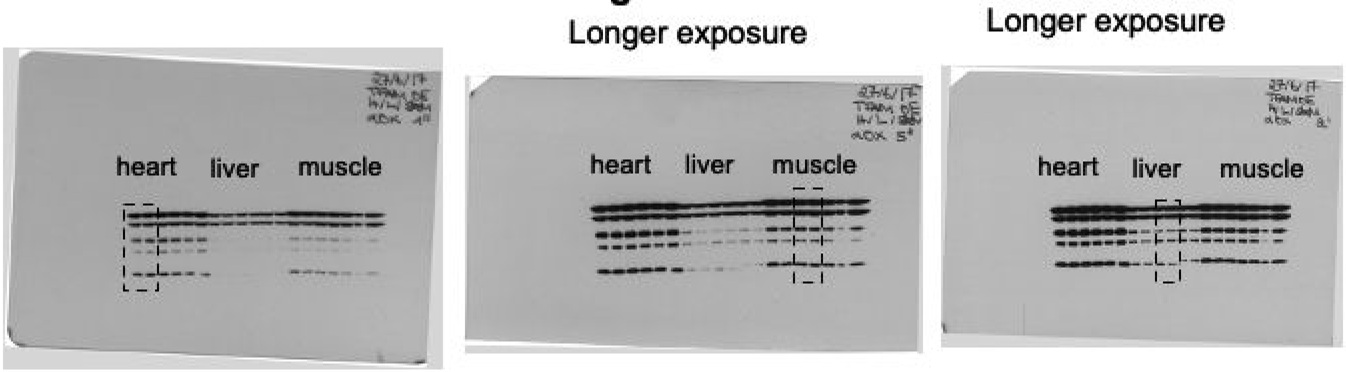

OXPHOS Cocktail

Figure 3A

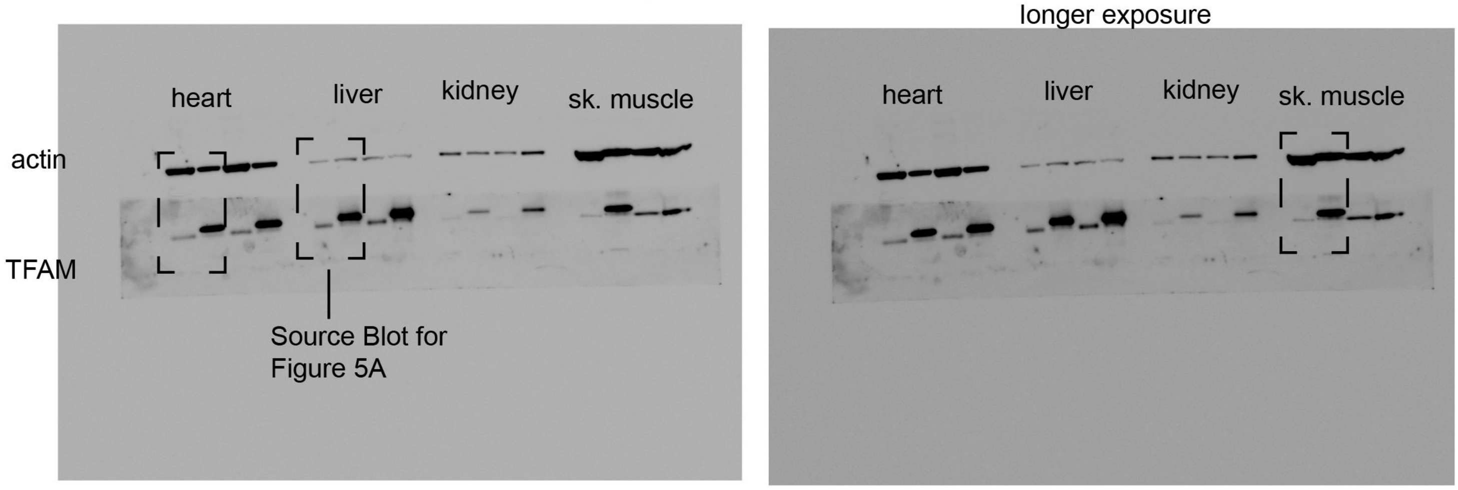

Figure 3D

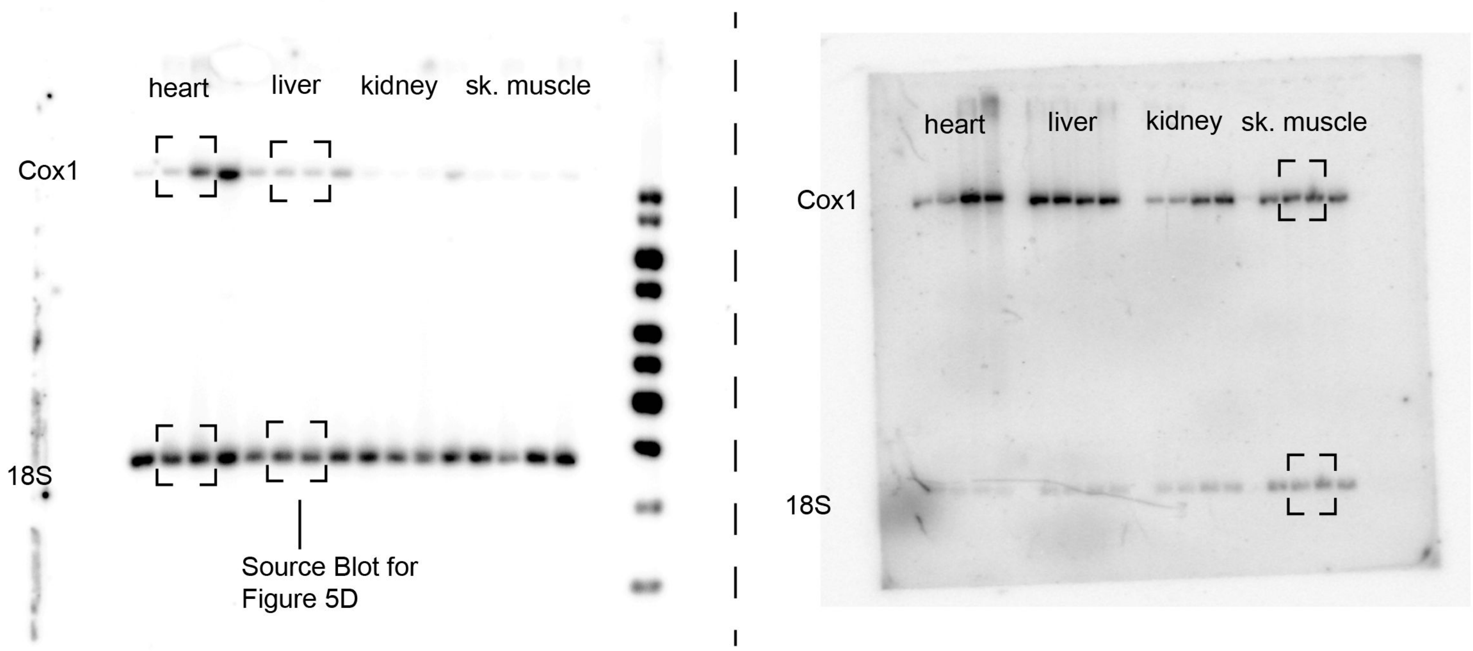

Figure 3F

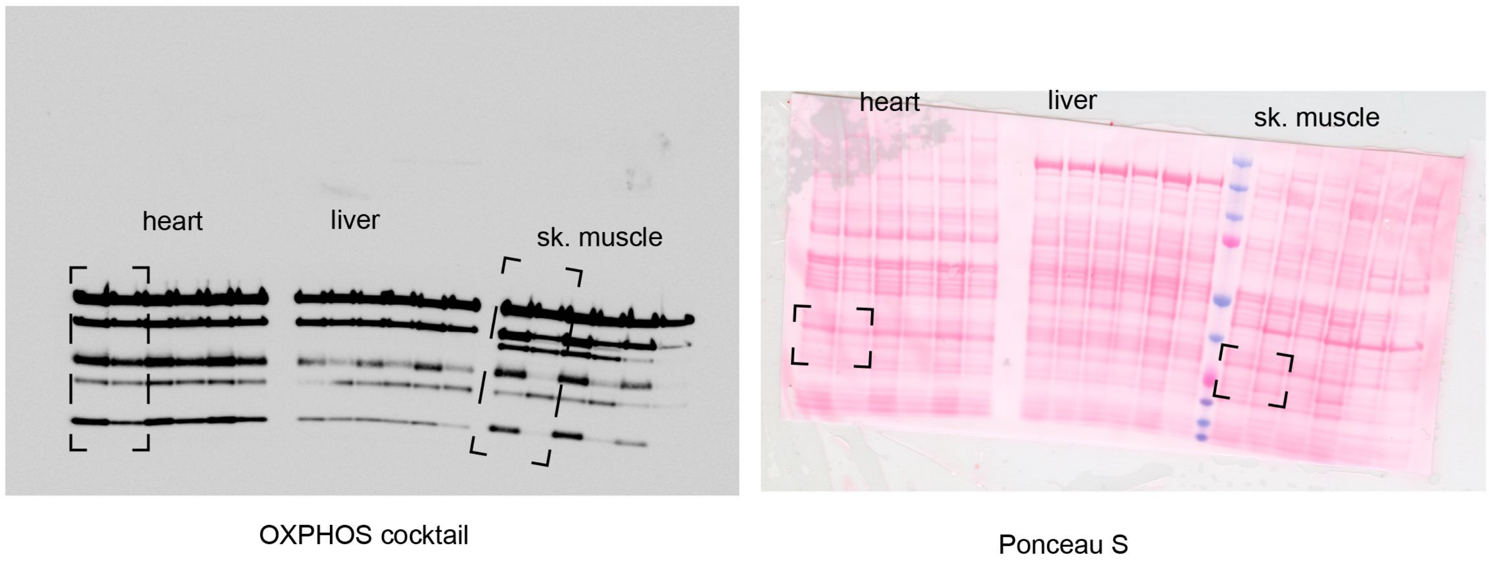

Figure 5A and D

PLEASE REFER TO SOURCE BLOTS FOR FIGURE 3

Figure 5F

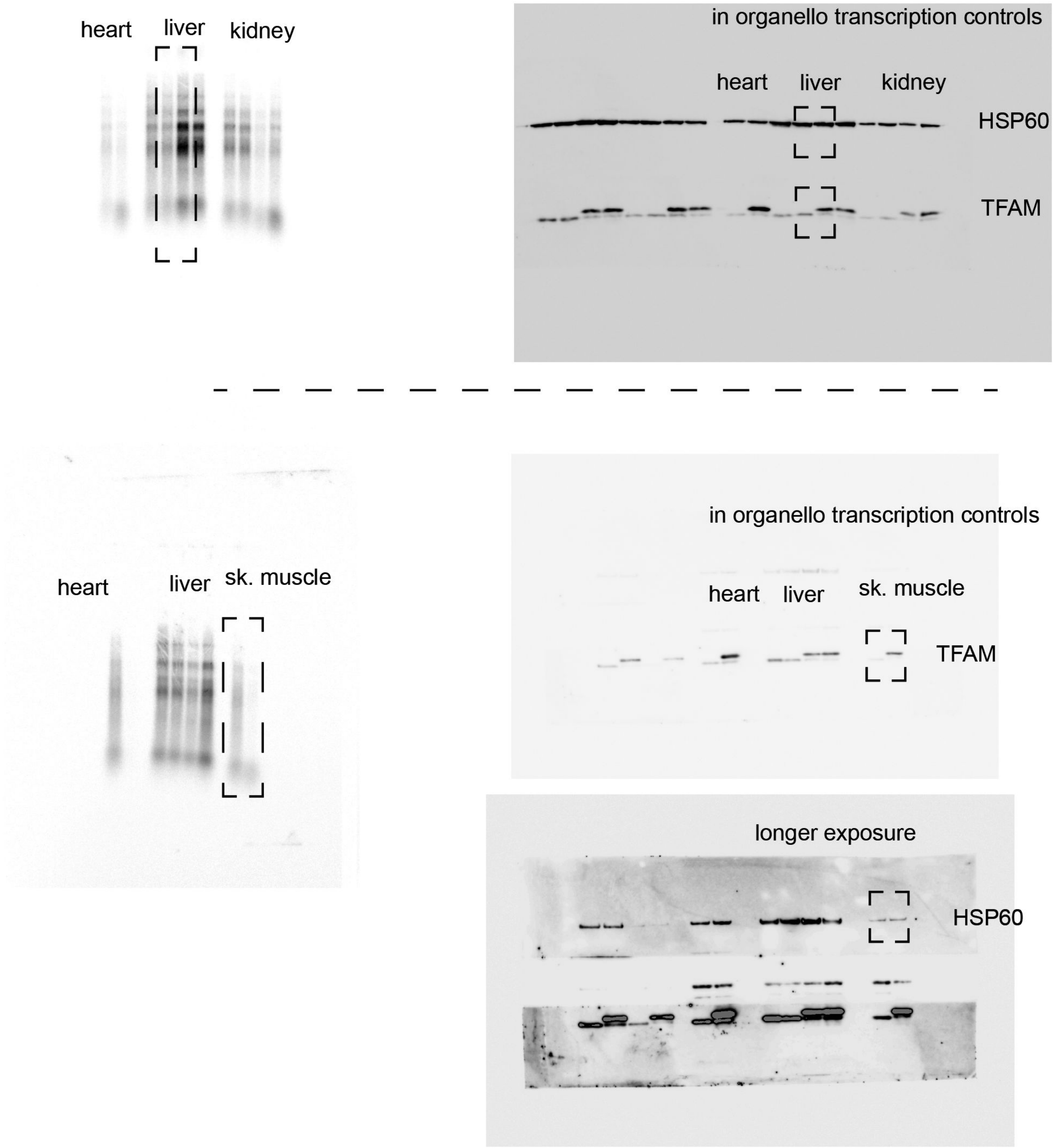

Figure S1A

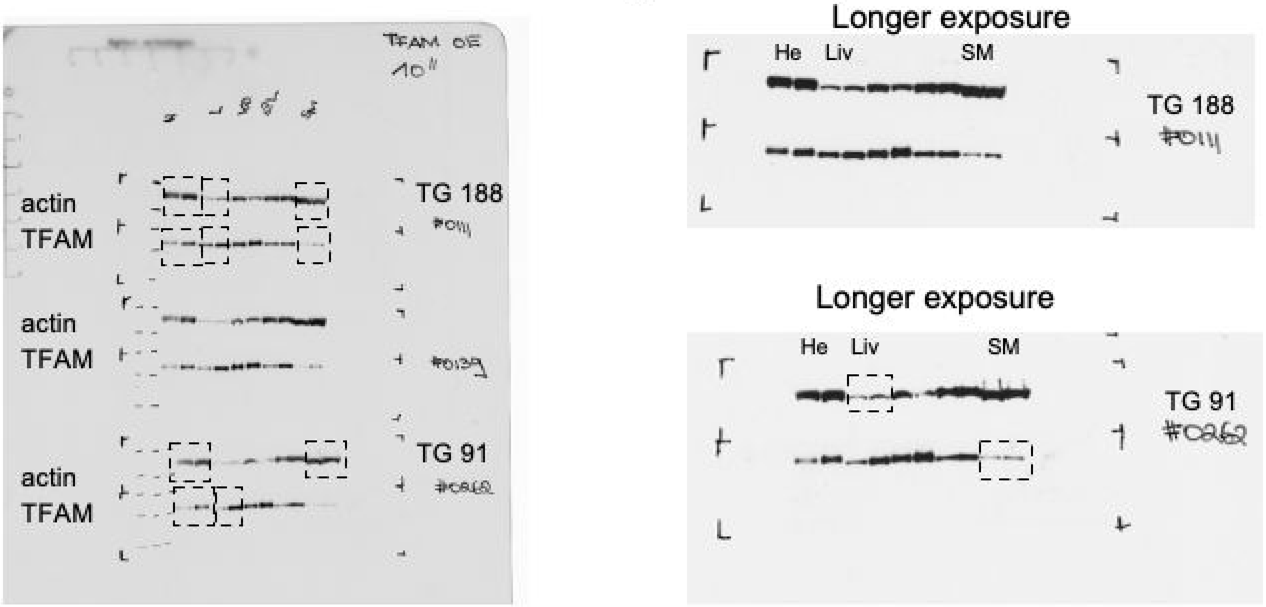

Figure S1D

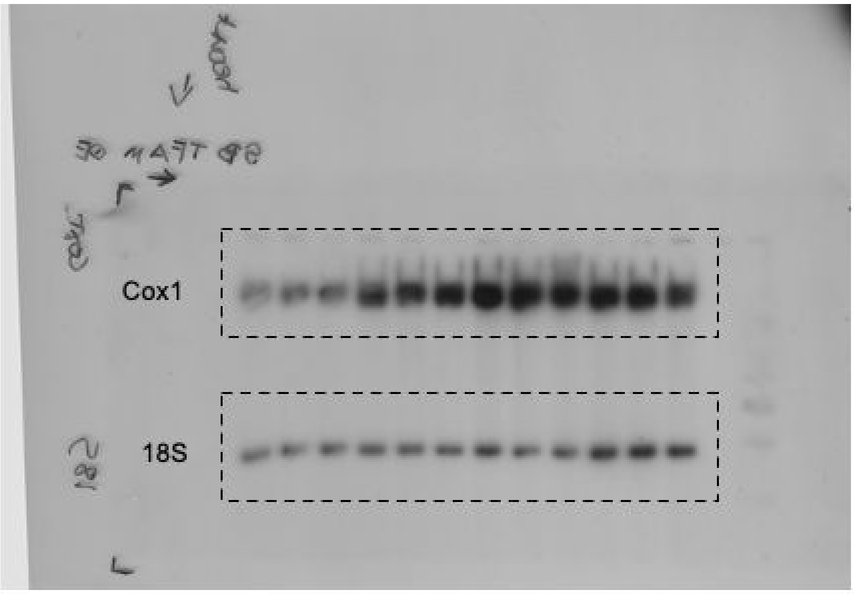

Figure S1E

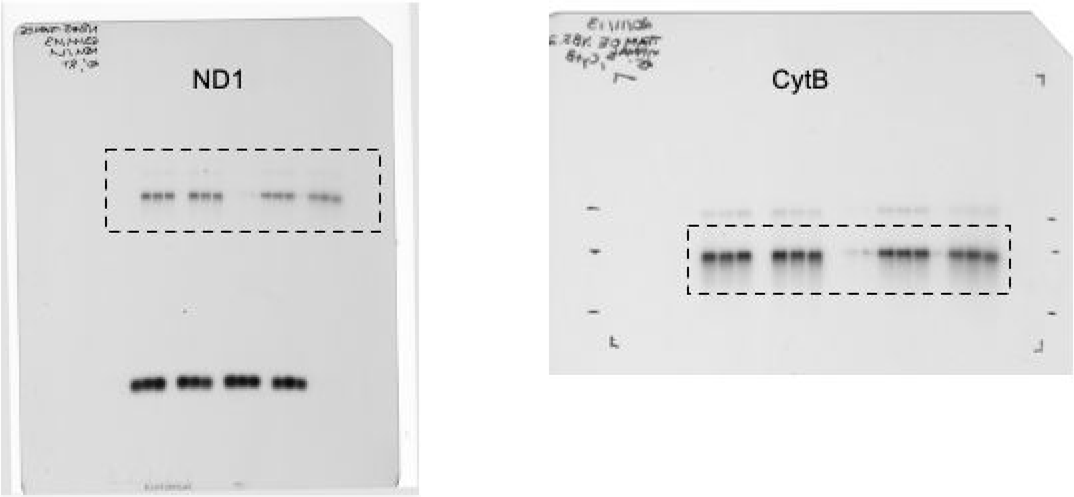

Figure S1E

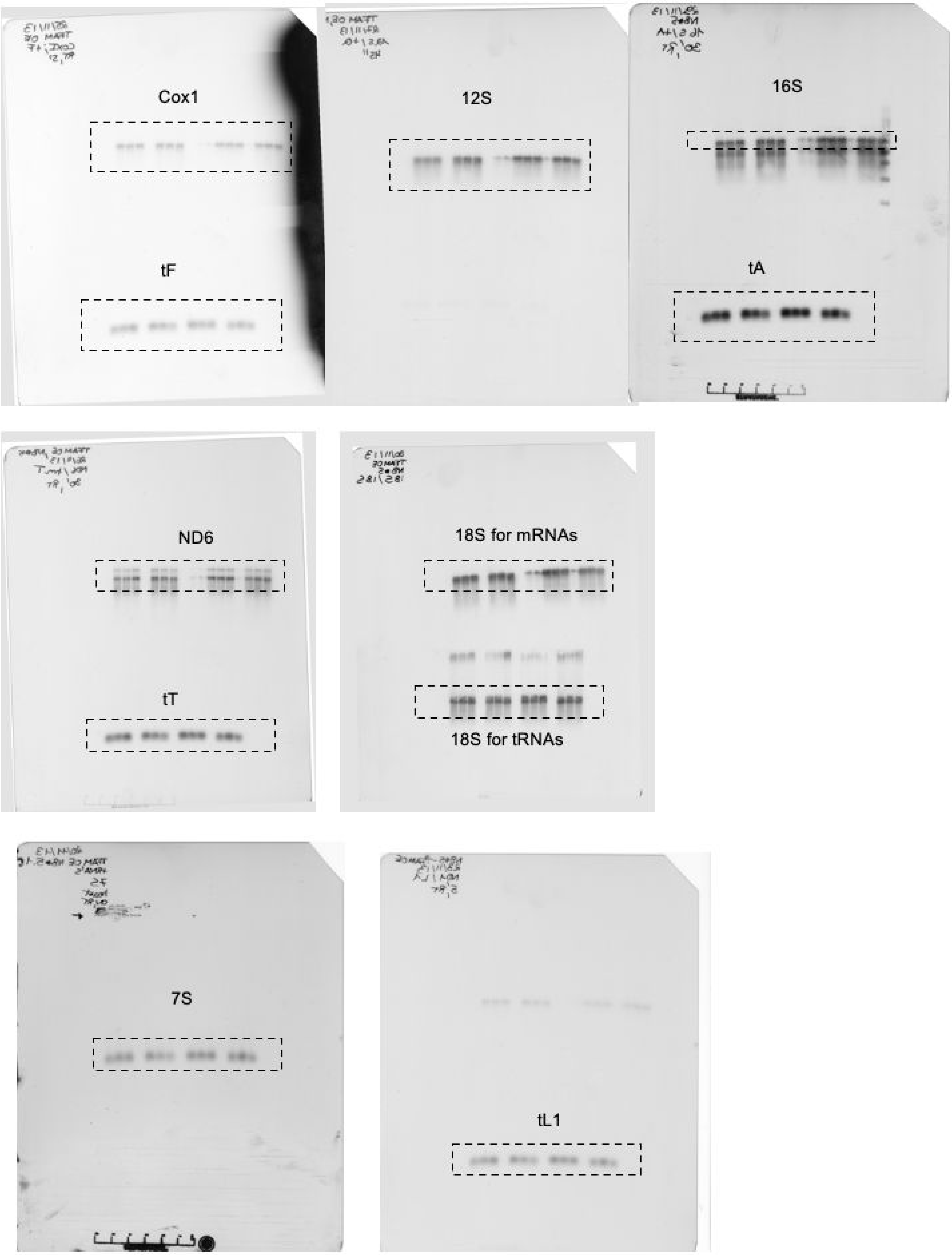

Figure S3D

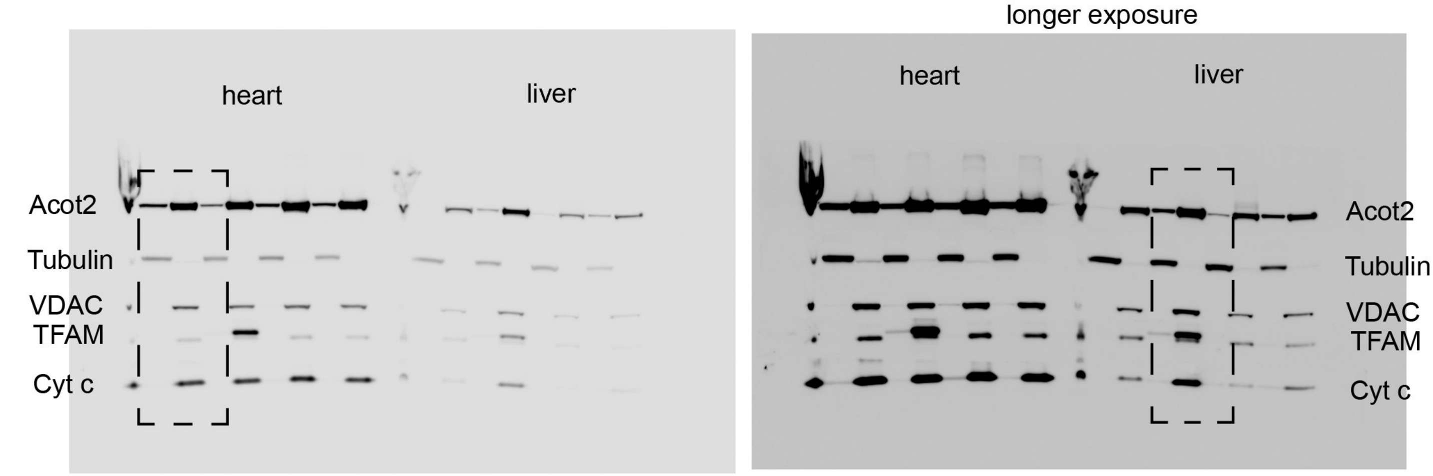

Figure S3E

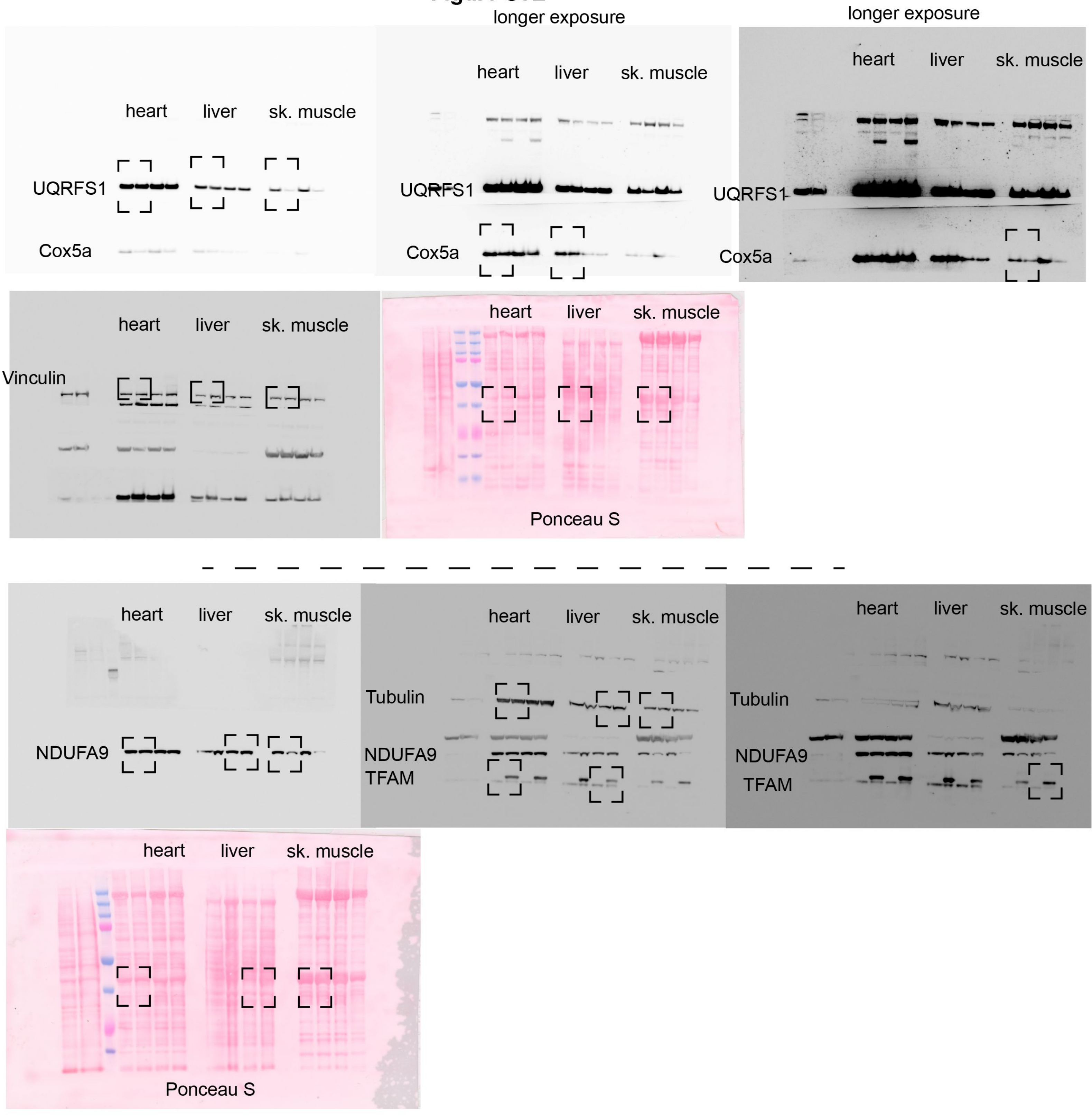

sk. muscle

Figure S5B

PhosTag Page

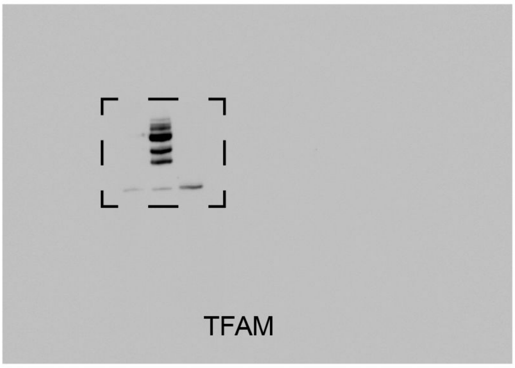

PhosTag Page

longer exposure in film

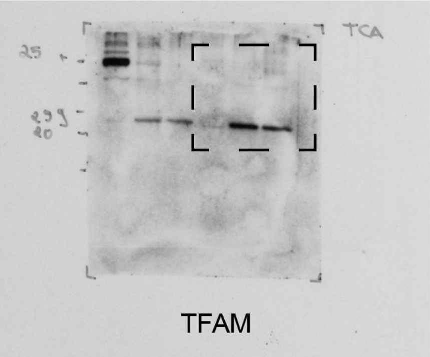

PhosTag Page

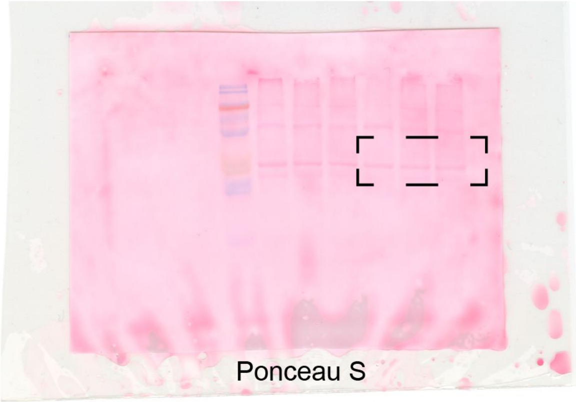

NuPage

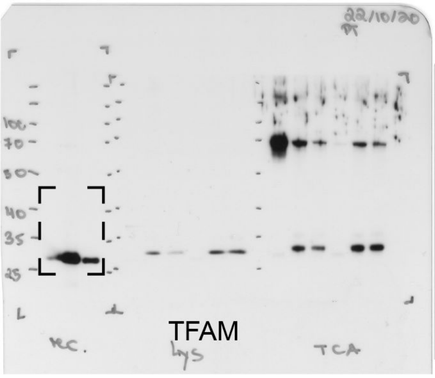

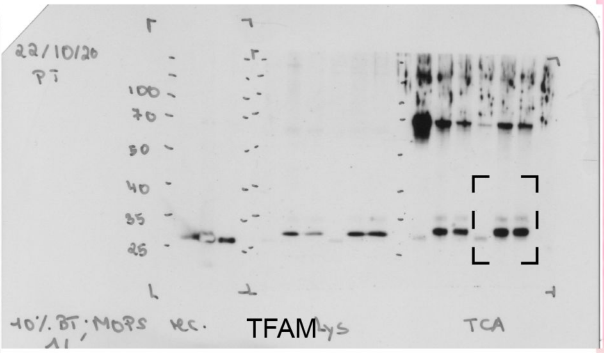

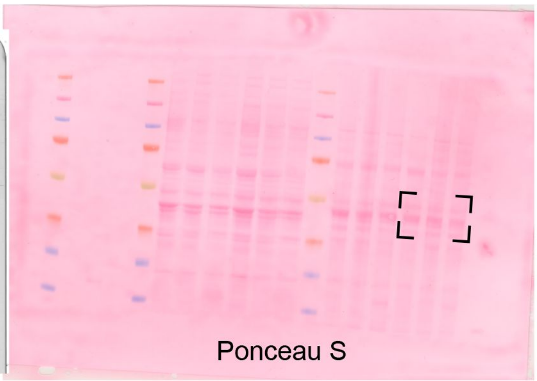

---

liver

Figure S5C

PhosTag Page

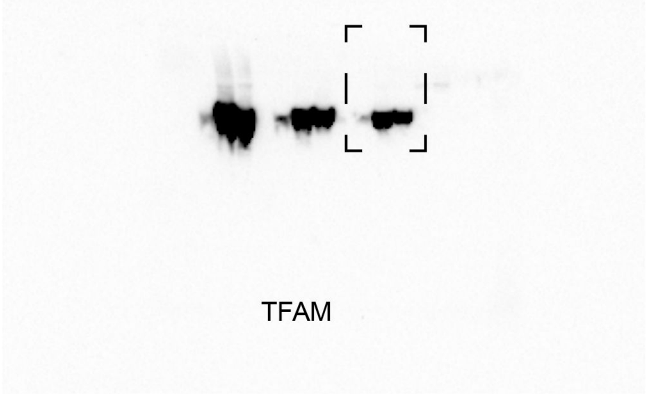

NuPage

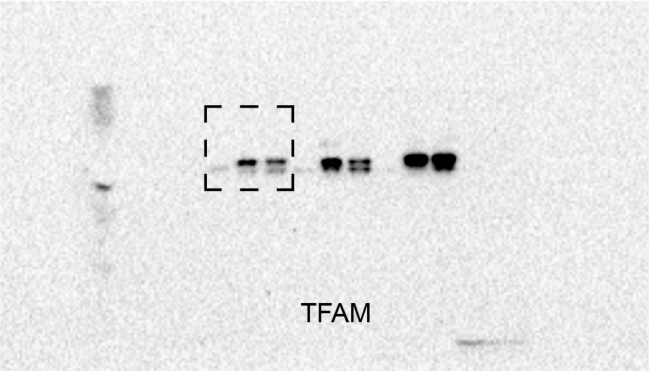

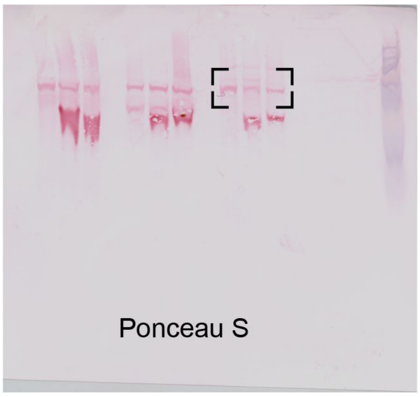

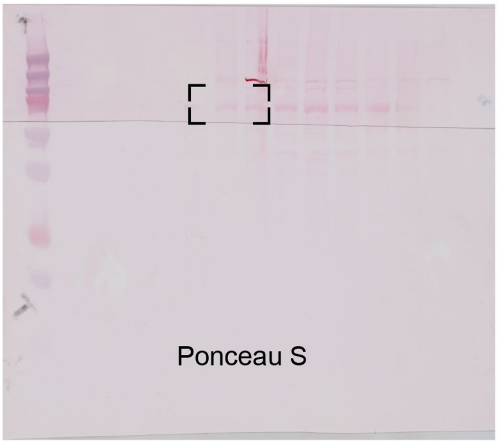

Supplement: Supplementary file 2 [file LSA-2021-01034_SdataF1B_F1D_F1F_F3A_F3D_F3F_F5A_F5D_F5F_FS1A_FS1D_FS1E_FS3D_FS3E_FS5B_FS5C.pdf]
